# Identification and validation of UBE2B as a prognostic biomarker promoting the development of esophageal carcinomas

**DOI:** 10.3389/fimmu.2024.1295305

**Published:** 2024-02-28

**Authors:** Han Ding, Jia-Cheng Xu, Zhi-Guo Ding, Lin-Feng Wu, Yan-Bo Liu, Yi-Fei Zhang, Tian-Yin Chen, Yi-Qun Zhang, Ping-Hong Zhou

**Affiliations:** ^1^ Department of Endoscopy Center and Endoscopy Research Institute, Zhongshan Hospital, Fudan University, Shanghai, China; ^2^ Department of Endoscopy, Shanghai Collaborative Innovation Center, Shanghai, China; ^3^ Endoscopy Center, Department of Gastroenterology, Shanghai East Hospital, School of Medicine, Tongji University, Shanghai, China; ^4^ Department of General Surgery, The Third People’s Hospital of Yangzhou, Yangzhou, Jiangsu, China

**Keywords:** bioinformatics, esophageal carcinoma, immune infiltration, prognostic biomarker, UBE2B

## Abstract

**Introduction:**

Ubiquitination is a crucial biological mechanism in humans, essential for regulating vital biological processes, and has been recognized as a promising focus for cancer therapy. Our objective in this research was to discover potential enzymes associated with ubiquitination that may serve as therapeutic targets for individuals with esophageal carcinoma (ESCA).

**Methods:**

To identify genes linked to the prognosis of ESCA, we examined mRNA sequencing data from patients with ESCA in the TCGA database. Further investigation into the role of the candidate gene in ESCA was conducted through bioinformatic analyses. Subsequently, we carried out biological assays to assess its impact on ESCA development.

**Results:**

Through univariate Cox regression analysis, we identified Ubiquitin Conjugating Enzyme E2 B (UBE2B) as a potential gene associated with the prognosis of ESCA. UBE2B exhibited significant upregulation and was found to be correlated with survival outcomes in ESCA as well as other cancer types. Additionally, UBE2B was observed to be involved in various biological pathways linked to the development of ESCA, including TNF-a signaling via NF-κB, epithelial-mesenchymal transition, inflammatory response, and hypoxia. Moreover, immune-related pathways like B cell activation (GO: 0042113), B cell receptor signaling pathway (GO: 0050853) and B cell mediated immunity (GO:0019724) were also involved. It was found that high expression of UBE2B was correlated with the increase of several kinds of T cells (CD8 T cells, Th1 cells) and macrophages, while effector memory T cell (Tem) and Th17 cells decreased. Furthermore, UBE2B showed potential as a prognostic biomarker for ESCA, displaying high sensitivity and specificity. Notably, proliferation and migration in ESCA cells were effectively suppressed when the expression of UBE2B was knocked down.

**Conclusions:**

To summarize, this study has made a discovery regarding the importance of gaining new insights into the role of UBE2B in ESCA. UBE2B might be an oncogene with good ability in predicting and diagnosing ESCA. Consequently, this discovery highlights the feasibility of targeting UBE2B as a viable approach for treating patients with ESCA.

## 1 Introduction

Esophageal carcinoma (ESCA), which encompasses esophagus squamous cell carcinoma (ESCC) and esophageal adenocarcinoma (ESAD), ranks eighth and poses a substantial threat to the global cancer burden (1). The global burden of ESCA was reflected in the high number of diagnoses in 2018, with over 572,000 patients being newly diagnosed, representing 3.2% of all newly diagnosed cancer cases worldwide (2). In spite of the disparities in the rates of occurrence among various geographical areas and ethnic backgrounds, there is a prevailing trend that indicates a progressive escalation in the incidence of ESCA (3–5). ESCA, a multifaceted ailment with intricate variations, is distinguished by numerous alterations in polymers and structural rearrangements (6, 7). The application of sequencing data analysis in the investigation of molecular targets relevant to the prognosis or development of ESCA has shown considerable promise.

Ubiquitination, a crucial protein degradation mechanism, serves as a vital component in human physiology. It plays a pivotal role in maintaining cellular homeostasis. Disturbed ubiquitination processes have been directly linked to numerous diseases, most notably cancer (8). Indeed, various studies have demonstrated the significant role of ubiquitination/deubiquitination processes in the development and progression of ESCA, ultimately contributing to its malignancy. Thus, directing attention towards molecules linked to the process of ubiquitination/deubiquitination presents a hopeful strategy for potentially efficacious treatment of ESCA. While previous research has predominantly concentrated on the impact of ubiquitin ligase enzyme (E3)/deubiquitination enzyme on the development of esophageal squamous cell carcinoma (ESCA) due to their remarkable substrate binding specificity, it is equally crucial to recognize the significance of ubiquitin-conjugating enzyme (E2) in the progression of cancer, including ESCA (9–11).

In a study that aimed to construct a competing endogenous RNA (ceRNA) network for esophageal adenocarcinoma (ESAD), Ubiquitin Conjugating Enzyme E2 B (UBE2B) was identified as a prognostic factor that could predict patient survival outcomes, implicating UBE2B as a potential marker for prognosis in ESAD (12). Therefore, attention has been drawn towards UBE2B, which has been observed to facilitate the progression of cancer in diverse types, including rectal, nasopharyngeal, and ovarian carcinoma (13–15). Although UBE2B was found to be related to overall survival (OS) in patients with ESCA based on mRNA sequencing data from The Cancer Genome Atlas Program (TCGA), its exact function and role in ESCA still need to be determined. Further studies are required to unravel the underlying mechanisms through which UBE2B impacts the progression of ESCA and to explore its potential as a therapeutic target for the disease.

By employing Cox regression analysis, we discerned a collection of ten genes linked to the OS of individuals diagnosed with ESCA, subsequently ordered by their respective p-values. Notably, UBE2B emerged as the sole gene related to ubiquitination within this grouping. Additionally, through bioinformatics analysis, distinctions were observed between ESCA patient groups categorized by varying levels of UBE2B expression. Ultimately, experimental findings substantiated the potential role of UBE2B within ESCA cells.

## 2 Materials and methods

### 2.1 Data collection

Among the entries in the TCGA database, there were a total of 185 ESCA patients for whom clinical information was available. Nonetheless, only 174 of these patients possessed mRNA sequencing data. Consequently, this study ultimately included 163 patient samples (including one duplicated sample) and 11 control samples. Prognostic data regarding these ESCA patients were procured from a previous investigation (16). Subsequent to the download, all mRNA sequencing data underwent normalization, resulting in transcripts per million (TPM) values. Prior to analysis, these TPM values were transformed to log2(TPM+1) for further exploration.

### 2.2 Prognostic genes selection

To explore the association between gene expression and prognosis in patients with ESCA, the cohort was divided into two groups based on the median expression value of each gene. An exploration into the association between the expression of each gene in these groups and the prognosis of ESCA patients was conducted via univariate Cox regression analyses. Subsequently, the obtained P-values were adjusted using the Benjamini and Hochberg (BH) method for correction. Protein-coding genes were then identified from the gene list, and the top ten genes were selected as candidate genes based on their ranking of P-values and hazard ratio (HR), with the most significant genes appearing at the top of the list and the least significant ones at the bottom.

### 2.3 The expression and prognostic value of UBE2B in pan-cancer

We examined the expression of UBE2B in various cancer types using mRNA sequencing data obtained from the TCGA pan-cancer database. Similar to the previous method mentioned, the data underwent processing. Subsequently, the sequencing data was merged with the corresponding clinical data for each specific cancer type. The log-rank test was then employed to evaluate the prognostic significance of UBE2B in these cancer types.

### 2.4 Differences between UBE2B expression groups in ESCA patients

The ESCA patients were divided into two groups, namely low-expression and high-expression, according to the median value of UBE2B expression. To identify genes that exhibited differential expression between these two groups, a differential gene expression analysis was performed using the DESeq2 R package (17). The selection criteria for identifying differently expressed genes (DEGs) were set as follows: | log2 fold change | ≥ 1 and an adjusted P value (adj. P) < 0.05.

Following the identification of DEGs based on the defined criteria, Gene Ontology (GO) and Kyoto Encyclopedia of Genes and Genomes (KEGG) analyses were conducted. These analyses aimed to elucidate the underlying molecular mechanisms and functional implications of the DEGs (18, 19).

In addition to the GO and KEGG analyses, Gene Set Enrichment Analysis (GSEA) was utilized to investigate the relationship between UBE2B expression and pathways. This approach aims to identify and assess the enrichment of predefined gene sets or pathways based on their correlation with UBE2B expression levels. By employing GSEA, the potential associations between UBE2B expression and various pathways can be explored and analyzed (19, 20). The gene set used for GSEA was acquired from the MSigDB collections database, which can be found at the following link: https://www.gsea-msigdb.org/gsea/msigdb/collections.jsp. This database contains a wide range of curated gene sets, including pathways, molecular signatures, and functional annotations, which can be utilized in GSEA analyses to assess the enrichment of these gene sets in relation to UBE2B expression.

To investigate immune infiltration in two groups, Single-Sample Gene Set Enrichment Analysis (ssGSEA) was conducted. ssGSEA evaluates the enrichment of specific gene signatures in each sample based on their gene expression profiles. It assigns a relative enrichment score to each sample, reflecting the activity or abundance of specific biological pathways or cell types within the sample (21). In this study, the Spearman’s correlation test was utilized to assess the correlation between UBE2B expression and immune cell infiltration patterns in patients with ESCA (22). This analysis allows researchers to explore the potential involvement of UBE2B in regulating immune cell infiltration in ESCA and provides insights into the immunological aspects of ESCA.

### 2.5 The prognostic value of UBE2B in ESCA

After consolidating the clinical data and UBE2B expression levels in ESCA patients, we conducted Cox regression analyses, both univariate and multivariate. Factors with P<0.1 in the univariate Cox regression analysis were subjected into the multivariate Cox regression analysis, and factors with P<0.05 in the multivariate Cox regression analysis were finally identified as independent prognostic factors for ESCA patients. Subsequently, these independent prognostic factors were employed in the development of a nomogram. To assess the accuracy and performance of the developed prognostic signature, a validation plot was generated. Additionally, we evaluated the diagnostic ability of UBE2B in ESCA by employing the receiver operating characteristic (ROC) curve.

### 2.6 Cell culture and RNA interference

Two esophageal cancer cell lines, KYSE-150 and ECA-109, were cultured under specific conditions. RPMI 1640 medium was used to culture KYSE-150 cells, while ECA-109 cells were cultured in DMEM. Both media were supplemented with 10% fetal bovine serum (FBS) and penicillin/streptomycin solution (100 U/ml). Then, the cells were incubated in a humidified carbon dioxide incubator at a temperature of 37°C.

Once the cells reached 50% confluency in the 6-well culture plates, we proceeded with the RNA interference procedure following the instructions provided by the manufacturer of lipofectamine 2000 (Thermo Fisher, 11668019). After 48 hours, total RNA and proteins were collected to assess the effectiveness of UBE2B gene silencing using quantitative reverse transcription polymerase chain reaction (RT-qPCR) and Western Blot techniques. The small interference RNA (siRNA) targeting UBE2B (siUBE2B) utilized in this study was synthesized by Genepharm Technologies (Shanghai, China). Two specific sequences were employed: siUBE2B-1 (GCAGTTATATTTGGACCAGAA) and siUBE2B-2 (CGGGATTTCAAGCGGTTACAA).

### 2.7 Cell viability, colony formation, and migration assays

To evaluate the viability of cells transfected with siUBE2B, we utilized the Cell Counting Kit-8 (CCK-8) reagent (Vazyme, A311-02-AA). Additionally, for the colony formation assay, UBE2B-silenced cells were seeded at a density of 1000 cells per well in 6-well culture plates and allowed to grow for approximately seven days. Subsequently, the cells were stained using a 0.1% (v/v) crystal violet solution.

For the migration assay, we used transwell chambers with a pore size of 8 mm (Corning,3422). The upper chamber was filled with 200 μl of serum-free media, while the lower chamber contained 500 μl of media supplemented with FBS (10% v/v). The cells were seeded in the upper chamber and allowed to migrate for 12 hours. Following this, the cells were fixed using a 4% (v/v) paraformaldehyde solution and subsequently stained with a 0.1% (v/v) crystal violet solution.

### 2.8 RT-qPCR and western blot

Total RNA extraction, reverse transcription, and real-time PCR were carried out using a commercial kit from Accurate Biotechnology (Changsha, Cat. No: AG21023, AG11706, and AG11718) following the manufacturer’s instructions. The primer sequences used for UBE2B and GAPDH were as follows: UBE2B Forward primer (5’-3’): TTGGACCAGAAGGGACACCT, UBE2B Reverse primer (5’-3’): CCTGGCTATTGGCTGGACTG; GAPDH Forward primer (5’-3’): GTCTCCTCTGACTTCAACAGCG, GAPDH Reverse primer (5’-3’): ACCACCCTGTTGCTGTAGCCAA.

Protein extraction and quantification were performed by commercial kit (Thermo Fisher Scientific; 78501 and 23227) following the kit’s instruction. Then, protein was uniformly loaded onto the SDS-PAGE gel (Shanghai Epizyme Biomedical Technology Co., Ltd; PG112). After about 90min, the protein was transferred to the nitrocellulose membrane (Millipore, HATF00010). Notably, the time of transference should be no more than 45 min. Subsequently, 5% non-fat milk was used to block the membrane at room temperature (1 hour). Finally, primary antibodies were employed to incubated the membrane at 4 °C overnight, and corresponding secondary antibodies were used to incubate at room temperature about 2 hours. The specific information about the antibodies were as the following: Anti-UBE2B (Abcam, ab128951, 1:1000), anti-GAPDH (Proteintech, 60004-1-Ig, 1:100000), secondary antibodies (ABclonal, AS014 and AS003,1:5000).

### 2.9 Statistical analysis

The quantitative data were represented as mean and median standard deviation (SD). At the same time, the qualitative data were presented as counts and percentage values. The count of colony formation and migration assays were calculated by ImageJ software. The results of biological assays were analyzed by t test, and p < 0.05 was regarded as statistically significant. All assays were repeated 3 times and showed the representative findings. The analyses were performed by R software and GraphPad Prism 8.0.

## 3 Results

### 3.1 High expression of UBE2B in most tumors correlates with adverse prognosis

**Figure 1:**
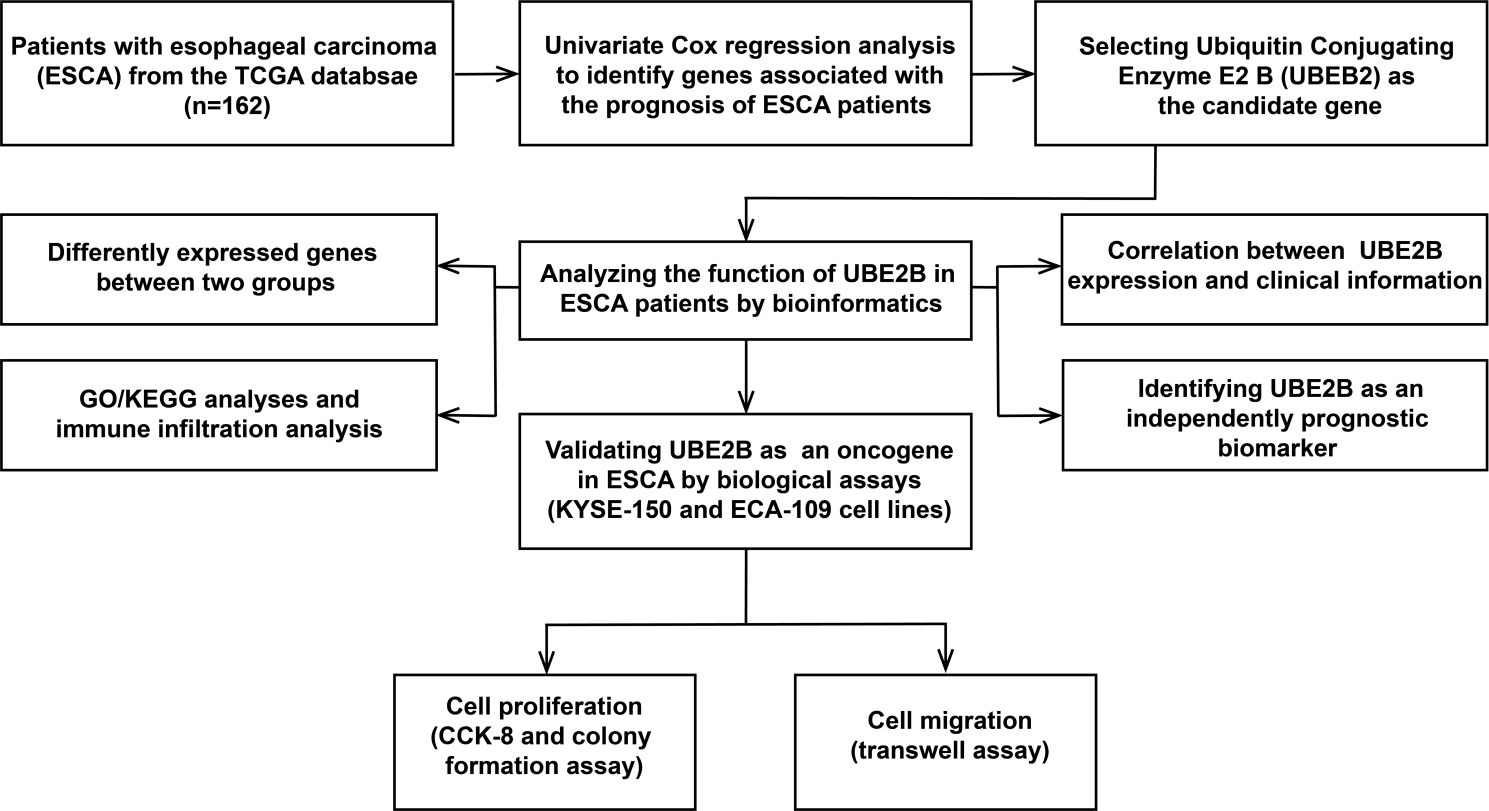
The flowchart of this study.

**Figure 2:**
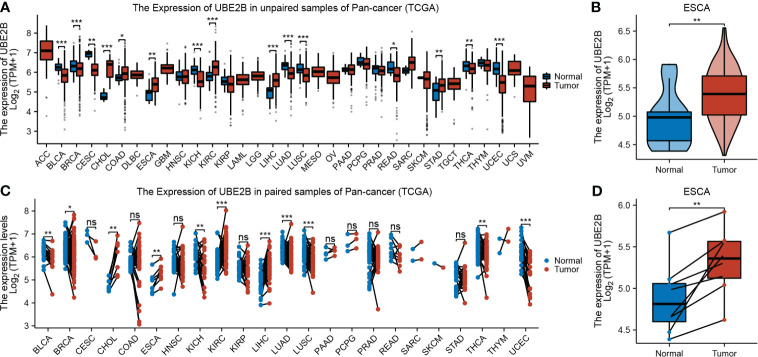
Expression of UBE2B in cancer. **(A, B)**. Expression of UBE2B in unpaired samples of patients with Pan-cancer and ESCA (Esophageal Carcinoma). **(C, D)**. Expression of UBE2B in paired samples of patients with Pan-cancer and ESCA (Esophageal Carcinoma). (*P < 0.05, **P < 0.01 and ***P < 0.001; ns, no significance)

**Figure 3:**
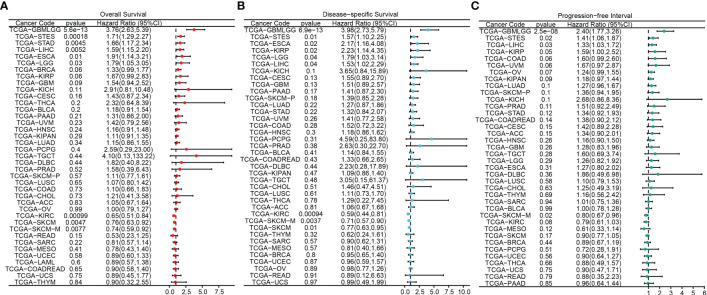
Association between UBE2B expression and prognosis in pan-cancers. **(A)** Overall survival analysis based on UBE2B expression. **(B)** Disease-specific survival analysis based on UBE2B expression. **(C)** Progression-free interval analysis based on UBE2B expression.

The flowchart of this study was shown in **Figure 1**. A sum of 601 genes encoding protein-coding related to the prognosis were detected (**Supplementary Table S1**). Out of these genes, UBE2B emerged as a noteworthy contender, ranking sixth due to its statistically significant low p-value as shown in **Table 1**, and fourth due to its considerably high hazard ratio as indicated in **Table 2**. The evidence strongly indicates the crucial involvement of UBE2B in the unfavorable prognosis of individuals with ESCA. It is worth mentioning that UBE2B stood out as the sole gene directly associated with ubiquitination, further enhancing its importance as a potential target of interest. Further investigations of UBE2B expression in unpaired samples of pan-cancer revealed diverse expression levels observed across different types of cancers. **Figures 2A, B** illustrated a notable increase in UBE2B expression in tumor samples obtained from various cancers, including CHOL, COAD, ESCA, KIRC, LIHC, and STAD. Similarly, in a paired pan-cancer analysis, UBE2B expression was found to be consistently high in the majority of tumor samples, including ESCA (**Figures 2C, D**). Furthermore, the expression of UBE2B was observed to be correlated with unfavorable prognosis in multiple tumor types, impacting both overall survival (OS) and disease-specific survival (DSS) (**Figure 3**).

**Table 1:** List of ten genes associated with the prognosis of ESCA patients (sorting by *P* value).

Gene name	statistic	HR	*P* value
KIAA0040	13.26000622	0.39407204	0.000389354
HSFX1	12.02562033	2.42819201	0.000837048
OXCT2	11.47500119	2.360161453	0.000979143
KCTD8	10.42570309	0.442599675	0.001513317
ZNF668	10.88345663	0.426111128	0.001546334
UBE2B	10.13856044	2.279471111	0.001585138
CFAP58	10.65385743	0.431310404	0.001646809
IGBP1	10.33470004	2.31983881	0.001765602
ABRACL	10.08301813	2.272988417	0.001775986

**Table 2:** List of ten genes associated with the prognosis of ESCA patients (sorting by HR).

Gene name	statistic	HR	*P* value
HSFX1	12.02562033	2.42819201	0.000837048
OXCT2	11.47500119	2.360161453	0.000979143
IGBP1	10.33470004	2.31983881	0.001765602
UBE2B	10.13856044	2.279471111	0.001585138
ABRACL	10.08301813	2.272988417	0.001775986
KCTD8	10.42570309	0.442599675	0.001513317
CFAP58	10.65385743	0.431310404	0.001646809
ZNF668	10.88345663	0.426111128	0.001546334
KIAA0040	13.26000622	0.39407204	0.000389354

### 3.2 Clinical characteristics

**Figure 4:**
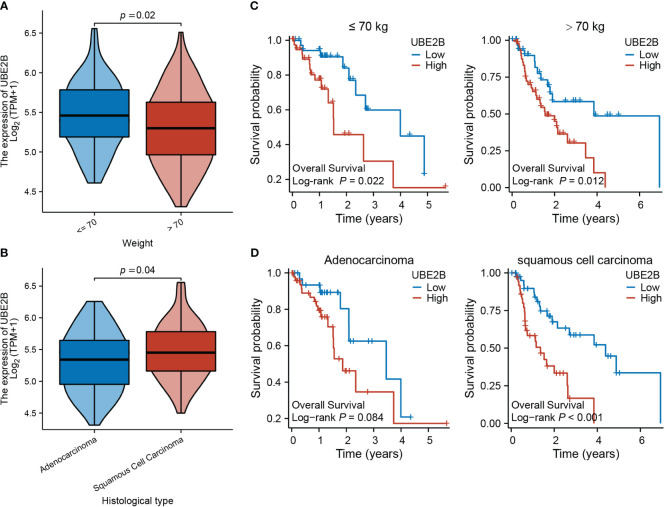
Expression of UBE2B in different clinical groups of patients with ESCA (Esophageal Carcinoma). **(A, B)** Differential UBE2B expression in different weight groups and histological types of ESCA patients. **(C, D)** The Kaplan-Meier (KM) plots showing the effect of UBE2B expression on patient survival indifferent weight and histological groups.

**Table 3** presents the clinical characteristics of 162 ESCA patients. It encompasses several factors including age, gender, race, height, weight, BMI, smoking status, alcohol history, presence of Barrett’s esophagus, history of reflux, presence of columnar mucosa dysplasia, presence of columnar metaplasia, tumor central location, TNM stage, pathologic stage, histological type, histologic grade, primary therapy outcome, presence of residual tumor, and administration of radiation therapy. The chi-squared test results demonstrated that the clinical characteristics of ESCA patients in the low and high expression groups of UBE2B did not have a statistically significant impact on the expression of UBE2B. Therefore, these findings suggest the importance of conducting further analysis to investigate the independent role of UBE2B in ESCA. Further analysis revealed significant differences in UBE2B expression based on weight and histological type (**Figures 4A, B**). Specifically, high expression of UBE2B was associated with poorer survival outcomes, especially in cases of squamous cell carcinoma (**Figures 4C, D**).

**Table 3:** Clinical characteristics of the patients.

Characteristics	Low expression of UBE2B (n=81)	High expression of UBE2B (n=81)	*P* value
Age, median (IQR)	61 (53, 72)	60 (54, 72)	0.951
Height, mean ± sd	173.16 ± 8.43	171.83 ± 8.55	0.336
Weight, median (IQR)	73 (63, 87.25)	70.5 (61, 83.25)	0.304
BMI, median (IQR)	24.5 (21.2, 28.0)	24.0 (21.3, 27.2)	0.676
Age (years)			0.753
≤ 60	40 (24.7%)	42 (25.9%)	
> 60	41 (25.3%)	39 (24.1%)	
Gender			0.822
Female	12 (7.4%)	11 (6.8%)	
Male	69 (42.6%)	70 (43.2%)	
Race			0.290
Non-white*	18 (12.5%)	25 (17.4%)	
White	52 (36.1%)	49 (34%)	
Height, (cm)			0.411
< 170	21 (13.7%)	26 (17%)	
≥ 170	55 (35.9%)	51 (33.3%)	
Weight, (kg)			0.527
≤ 70	36 (22.5%)	40 (25%)	
> 70	44 (27.5%)	40 (25%)	
BMI			0.376
≤ 25	39 (25.5%)	45 (29.4%)	
> 25	37 (24.2%)	32 (20.9%)	
Smoker			0.906
No	23 (16%)	25 (17.4%)	
Yes	47 (32.6%)	49 (34%)	
Alcohol history			0.903
No	24 (15.1%)	23 (14.5%)	
Yes	56 (35.2%)	56 (35.2%)	
Barrett esophagus			0.376
No	55 (41.7%)	51 (38.6%)	
Yes	16 (12.1%)	10 (7.6%)	
Reflux history			0.308
No	39 (28.7%)	46 (33.8%)	
Yes	28 (20.6%)	23 (16.9%)	
Columnar mucosa dysplasia			0.652
Negative/ no dysplasia	20 (29%)	18 (26.1%)	
Low/High grade dysplasia	18 (26.1%)	13 (18.8%)	
Columnar metaplasia			0.559
No	36 (36.7%)	33 (33.7%)	
Yes	17 (17.3%)	12 (12.2%)	
Tumor cental location			0.639
Mid and Proximal	22 (13.7%)	25 (15.5%)	
Distal	58 (36%)	56 (34.8%)	
Pathologic T stage			0.373
T1 and T2	34 (23.4%)	30 (20.7%)	
T3 and T4	37 (25.5%)	44 (30.3%)	
Pathologic N stage			0.247
N0	36 (25%)	30 (20.8%)	
N1, N2 and N3	35 (24.3%)	43 (29.9%)	
Pathologic M stage			1.000
M0	59 (45.7%)	62 (48.1%)	
M1	4 (3.1%)	4 (3.1%)	
Pathologic stage			0.205
Stage I and II	45 (31.7%)	40 (28.2%)	
Stage III and IV	24 (16.9%)	33 (23.2%)	
Histological type			0.432
Adenocarcinoma	43 (26.5%)	38 (23.5%)	
Squamous Cell Carcinoma	38 (23.5%)	43 (26.5%)	
Histologic grade			0.991
G1 and G2	40 (31.5%)	42 (33.1%)	
G3	22 (17.3%)	23 (18.1%)	
Primary therapy outcome			1.000
CR, PR and SD	41 (44.1%)	43 (46.2%)	
PD	4 (4.3%)	5 (5.4%)	
Residual tumor			0.381
R0	59 (44%)	62 (46.3%)	
R1 and R2	8 (6%)	5 (3.7%)	
Radiation therapy			0.441
No	56 (38.9%)	52 (36.1%)	
Yes	16 (11.1%)	20 (13.9%)	
OS event			0.149
Alive	53 (32.7%)	44 (27.2%)	
Dead	28 (17.3%)	37 (22.8%)	
DSS event			0.273
No	61 (37.9%)	54 (33.5%)	
Yes	20 (12.4%)	26 (16.1%)	
PFI event			0.637
No	40 (24.7%)	43 (26.5%)	
Yes	41 (25.3%)	38 (23.5%)	

### 3.3 Functional enrichment analysis of differentially expressed UBE2B in ESCA

**Figure 5:**
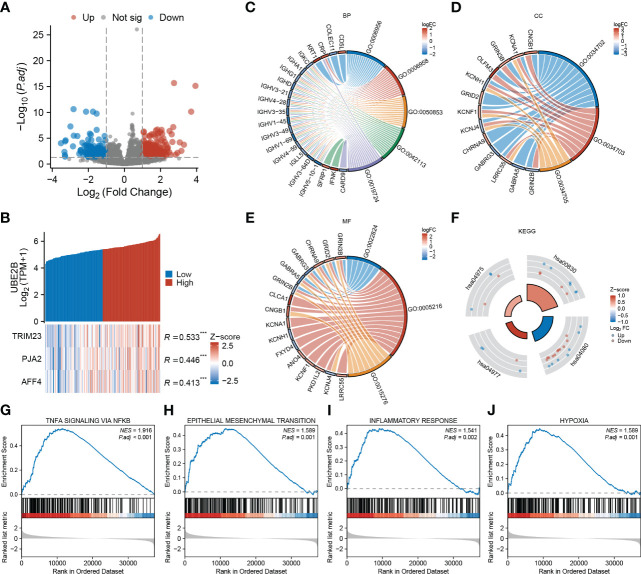
Functional enrichment analysis based on UBE2B expression in patients with ESCA (Esophageal Carcinoma). **(A)** Volcano plot depicting differentially expressed genes based on UBE2B expression. **(B)** Prediction of the E3 ligase that interacts with UBE2B. **(C–F)**. Gene Ontology (GO) and Kyoto Encyclopedia of Genes and Genomes (KEGG) analyses. **(G–J)**. Gene Set Enrichment Analysis (GSEA) based on UBE2B expression.

After applying the selection criteria, a total of 713 differentially expressed genes (DEGs) were identified in ESCA. These consisted of 488 up-regulated genes and 225 down-regulated genes, as depicted in **Figure 5A**. Interestingly, among these DEGs, three E3 ligases (TRIM23, PJA2, and AFF4) showed a positive correlation with UBE2B expression, suggesting potential interactions between these E3 ligases and UBE2B in the ubiquitination process (**Figure 5B**).

Additionally, GO enrichment analyses unveiled that UBE2B expression was linked to various immune-related biological processes (BP), including complement activation (GO:0006956 and GO:0006958), B cell-mediated immunity (GO:0050853, GO:0042113, GO:0019724) and humoral immune response (GO:0006959) (**Figure 5C**). Additionally, ion channel-related cellular components (CC) including ion channel complex (GO:0034702), cation channel complex (GO:0034703) and potassium channel complex (GO:0034705), as well ion channel- related molecular functions (MF) such as transmitter-gated ion channel activity (GO:0022824), ion channel activity (GO:0005216) and ligand-gated ion channel activity (GO:0015276) were also enriched (**Figures 5D, E**). Furthermore, KEGG analyses indicated that UBE2B-associated DEGs might participate in some processes (**Figure 5F**) like fat digestion and absorption (hsa04975), retinol metabolism (hsa00830), neuroactive ligand-receptor interaction (hsa04080) and vitamin digestion and absorption (hsa04977). Utilizing gene sets from the MSigDB collection (h.all.v7.5.1. symbols [Hallmarks] (50)), several signaling pathways were found to be enriched, including TNF-a signaling via NF-κB, epithelial-mesenchymal transition (EMT), inflammatory response and hypoxia (**Figures 5G–J**). These results suggested that UBE2B could affect the development of ESCA by regulating the immune response, ion channel, and some biological pathways.

**Figure 6:**
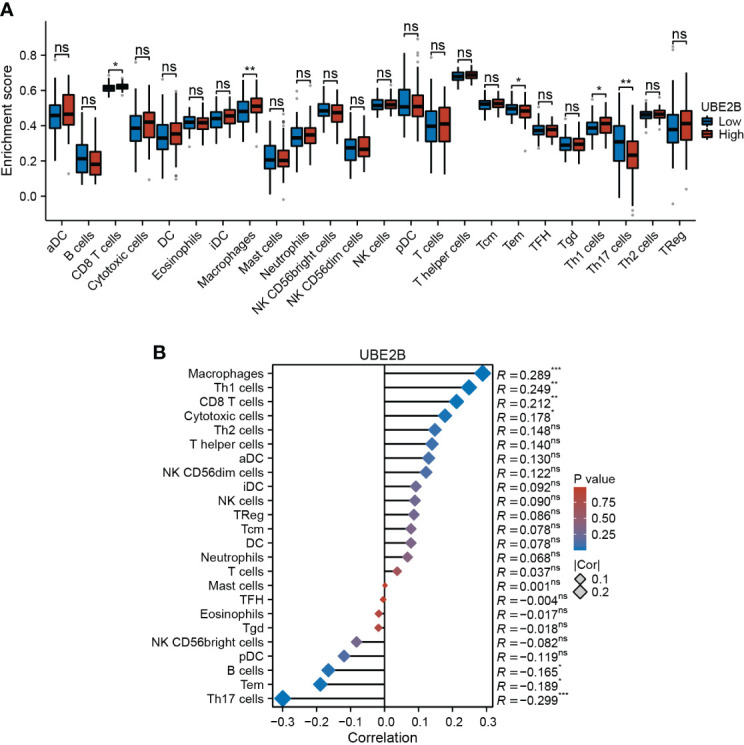
Immune infiltration in two UBE2B expression groups. **(A)** The different immune cells are in two groups. **(B)** Correlation analysis between immune cell types and UBE2B expression.

Furthermore, based on immune infiltration scores, the infiltration levels of CD8 T cells, macrophages, effector memory T cell (Tem), Th1 cells and Th17 cells were significantly different in low- and high-expression of UBE2B groups (**Figure 6A**). Additionally, the expression of UBE2B was found to be correlated with macrophages, Th1 cells, CD8 T cells, cytotoxic cells, B cells, Tem and Th17 cells (**Figure 6B**).

### 3.4 UBE2B as a potential prognostic and diagnostic biomarker for patients with ESCA

**Figure 7:**
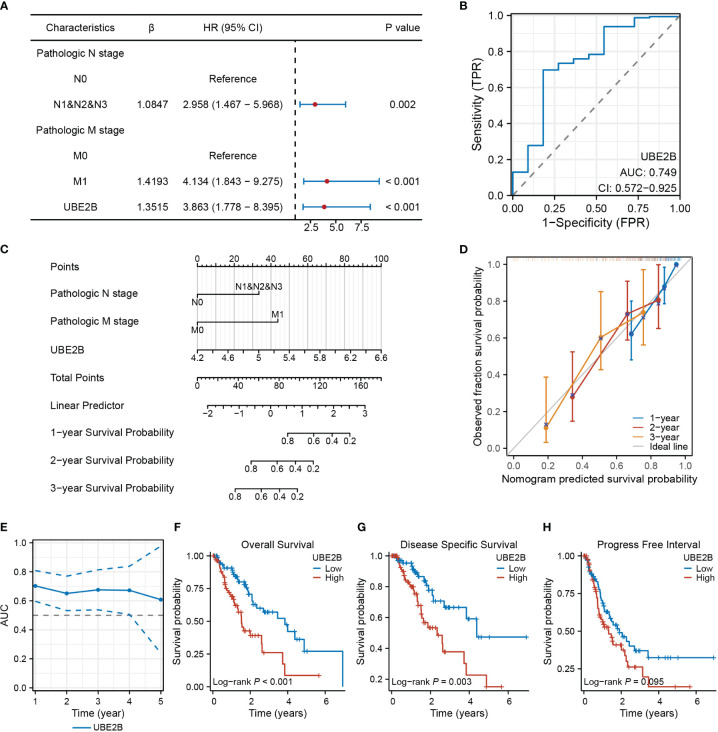
UBE2B as a biomarker in patients with ESCA (Esophageal Carcinoma). **(A)** Forest plot showing factors related to the prognosis of patients with ESCA (Esophageal Carcinoma). **(B)** Receiver Operating Characteristic (ROC) curve assessing the diagnostic ability of UBE2B in ESCA (Esophageal Carcinoma). **(C)** Nomogram for predicting the survival probability of patients with ESCA (Esophageal Carcinoma). **(D)** Calibration plot of the predictive performance of nomogram. **(E)** UBE2B evaluation in predicting the prognosis of patients with ESCA (Esophageal Carcinoma). **(F-H)**. The Kaplan-Meier (KM) plots showing the effect of UBE2B expression on the survival outcomes of patients with ESCA (Esophageal Carcinoma), including overall survival, disease specific survival and progress free interval, respectively.

The results of univariate and multivariate Cox regression analyses identified several prognostic factors (**Table 4**), including pathologic N stage (HR: 6.675, 95% CI: 1.362 - 32.709, P=0.019), pathologic M stage (HR: 29.443, 95% CI: 1.842 - 470.748, P=0.017) and the expression of UBE2B (HR: 4.990, 95% CI: 1.101 - 22.623, P=0.037). Subsequently, a multivariate Cox regression analysis was performed based on these three prognostic factors. The results are illustrated in the forest plot (**Figure 7A**). Moreover, the ROC curve demonstrated that UBE2B exhibited good sensitivity and specificity in diagnosing ESCA (AUC: 0.749, 95%CI: 0.572-0.925), indicating that UBE2B could be a diagnostic biomarker for patients with ESCA (**Figure 7B**). Based on the results from **Figure 7A**, a nomogram was constructed to predict the 1-, 2- and 3-year survival probabilities of patients with ESCA and performance was assessed using calibration plots (**Figures 7C, D**). In addition, the time-dependent ROC curve further showed the capability of UBE2B in predicting the OS of patients with ESCA (**Figure 7E**). Furthermore, the log-rank test revealed that UBE2B was significantly correlated with the OS (*P*<0.001) and DSS (*P*=0.003) of patients with ESCA, but not progression-free interval (**Figures 7F–H**). These findings establish that UBE2B was an independent prognostic factor for patients with ESCA.

**Table 4:** The Cox regression analyses in ESCA patients (OS).

Characteristics	N	Univariate analysis		Multivariate analysis	
		Hazard ratio (95% CI)	*P*	Hazard ratio (95% CI)	*P*
Age (years)			0.618		
≤ 60	82	Reference			
> 60	80	0.882 (0.538 - 1.446)	0.619		
Gender			0.045		
Female	23	Reference		Reference	
Male	139	2.304 (0.921 - 5.763)	0.075	39090896 (0.00 - Inf)	0.997
Race			0.705		
Non-white*	43	Reference			
White	101	1.147 (0.560 - 2.349)	0.708		
Height (cm)			0.253		
< 170	47	Reference			
≥ 170	106	1.433 (0.757 - 2.713)	0.269		
Weight (kg)			0.161		
≤ 70	76	Reference			
> 70	84	1.439 (0.859 - 2.408)	0.167		
BMI			0.862		
≤ 25	84	Reference			
> 25	69	1.047 (0.623 - 1.760)	0.862		
Smoker			0.297		
No	48	Reference			
Yes	96	1.394 (0.736 - 2.640)	0.309		
Alcohol history			0.167		
No	47	Reference			
Yes	112	0.695 (0.419 - 1.154)	0.160		
Barrett esophagus			0.439		
No	106	Reference			
Yes	26	1.266 (0.703 - 2.281)	0.432		
Reflux history			0.918		
No	85	Reference			
Yes	51	0.971 (0.550 - 1.712)	0.918		
Columnar mucosa dysplasia			0.512		
Negative/no	38	Reference			
Low/High grade	31	1.246 (0.646 - 2.403)	0.512		
Columnar metaplasia			0.496		
No	69	Reference			
Yes	29	1.227 (0.685 - 2.199)	0.492		
Tumor cental location			0.643		
Mid & Proximal	47	Reference			
Distal	114	1.166 (0.605 - 2.247)	0.647		
Pathologic T stage			0.332		
T1 and T2	64	Reference			
T3 and T4	81	1.312 (0.756 - 2.277)	0.334		
Pathologic N stage			< 0.001		
N0	66	Reference		Reference	
N1, N2 and N3	78	2.970 (1.606 - 5.493)	< 0.001	6.675 (1.362 - 32.709)	0.019
Pathologic M stage			< 0.001		
M0	121	Reference		Reference	
M1	8	5.075 (2.312 - 11.136)	< 0.001	29.443 (1.842 - 470.748)	0.017
Pathologic stage			< 0.001		
Stage I and II	85	Reference		Reference	
Stage III and IV	57	3.223 (1.807 - 5.747)	< 0.001	0.427 (0.105 - 1.738)	0.235
Histological type			0.448		
Adenocarcinoma	81	Reference			
Squamous Cell Carcinoma	81	0.821 (0.493 - 1.370)	0.451		
Histologic grade			0.089		
G1 and G2	82	Reference		Reference	
G3	45	1.626 (0.935 - 2.827)	0.085	1.085 (0.219 - 5.374)	0.921
Primary therapy outcome			0.020		
SD, PR and CR	84	Reference		Reference	
PD	9	3.576 (1.349 - 9.478)	0.010	0.626 (0.111 - 3.533)	0.596
Residual tumor			0.025		
R0	121	Reference		Reference	
R1 and R2	13	2.420 (1.191 - 4.919)	0.015	1.357 (0.085 - 21.699)	0.829
Radiation therapy			0.549		
No	108	Reference			
Yes	36	0.804 (0.388 - 1.667)	0.558		
UBE2B	162	2.605 (1.432 - 4.737)	0.002	4.990 (1.101 - 22.623)	0.037

### 3.5 Enhanced UBE2B activity stimulates ESCA cell proliferation and migration

**Figure 8:**
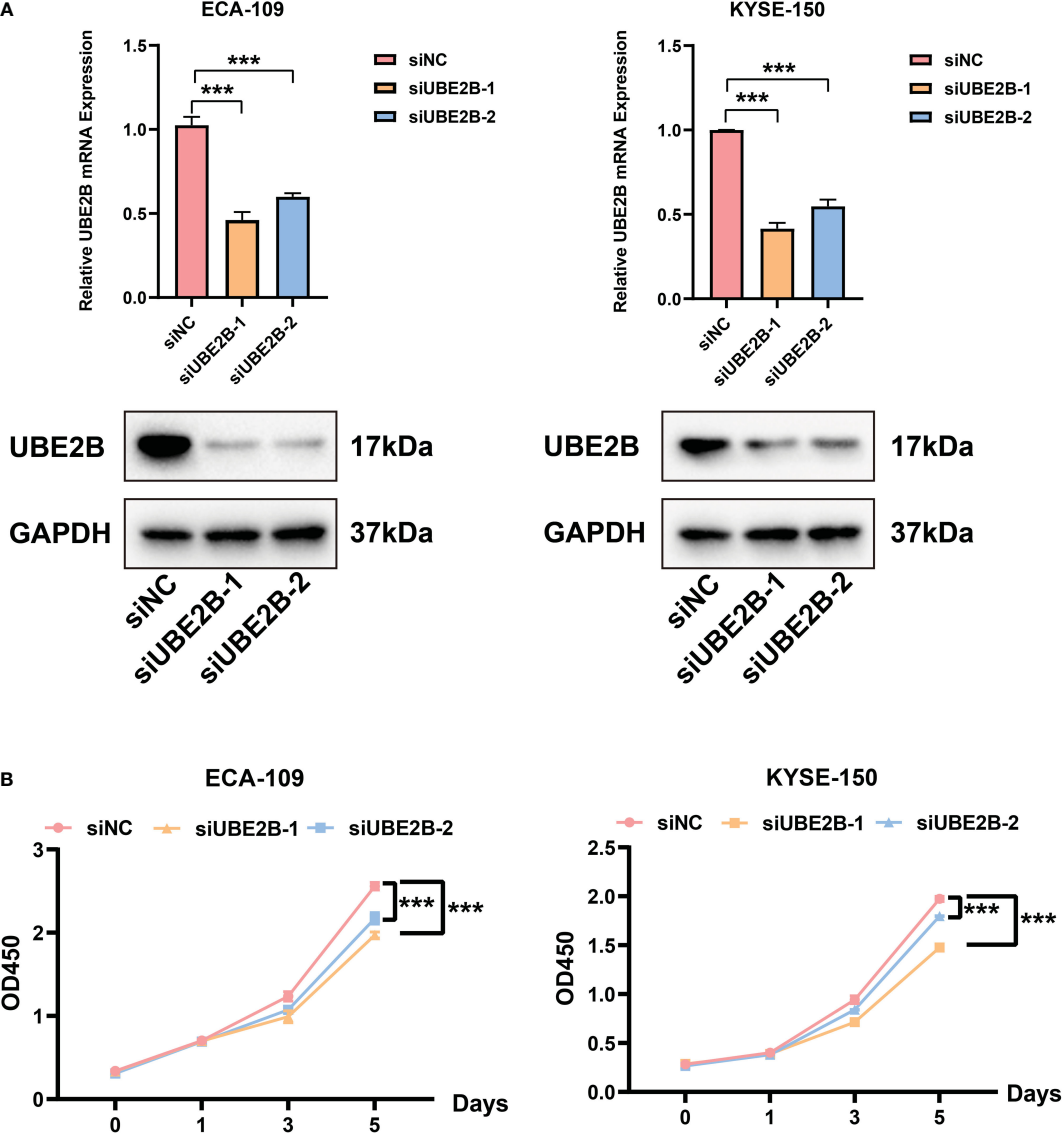
Silencing UBE2B suppressed the proliferation of ECA-109 and KYSE-150 cells. **(A)** Assessment of silencing efficacy of UBE2B. **(B)** CCK-8 assay to measure cell proliferation. (***P < 0.001).

**Figure 9:**
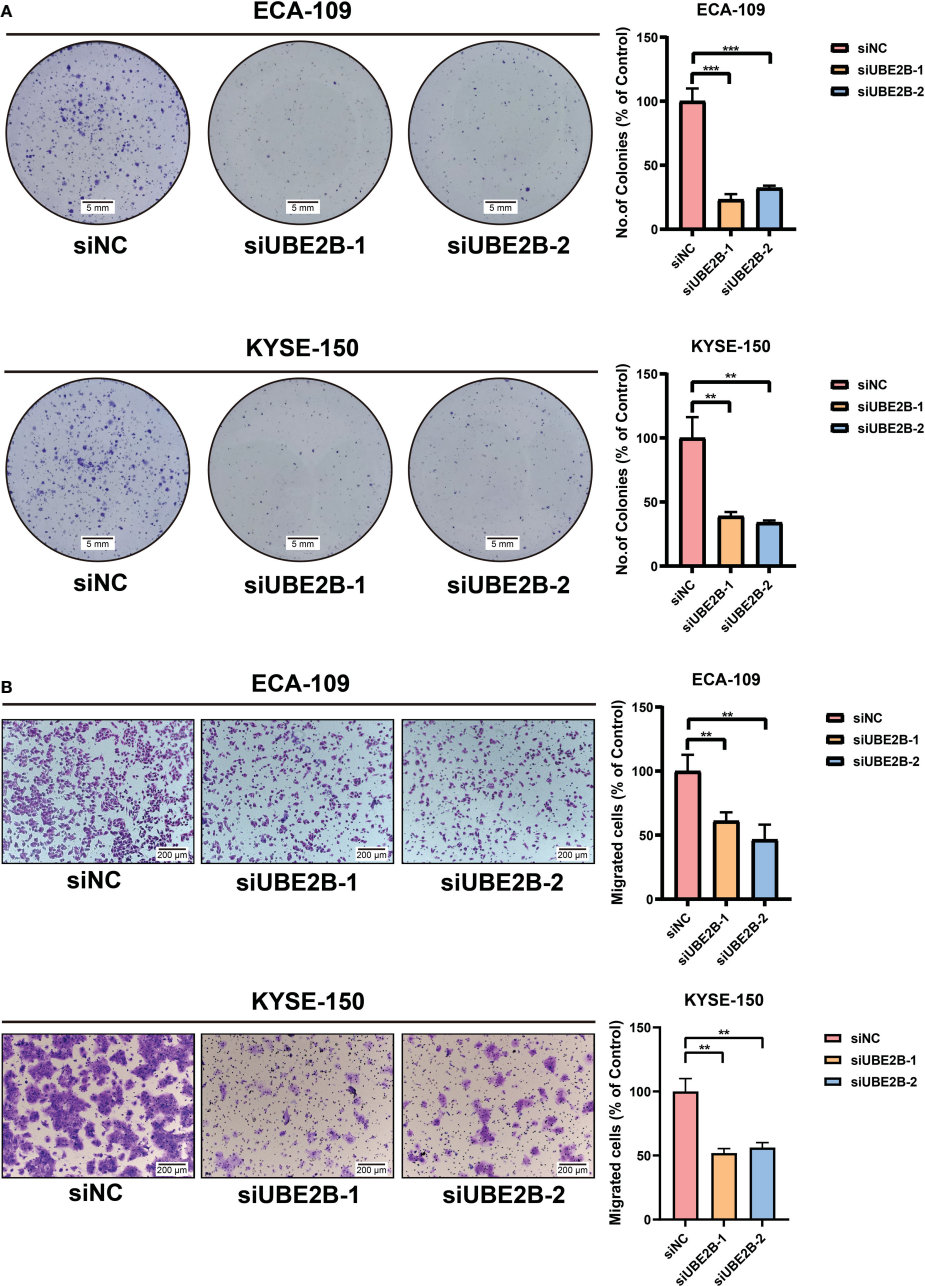
Silencing UBE2B inhibited the growth and migration of ECA-109 and KYSE-150 cells. **(A)** Colony formation assay to investigate cell growth ability. **(B)** Detection of cell migration using a migration assay. (**P < 0.01, ***P < 0.001).

The RT-qPCR and western blot were used to detect the silencing efficacy of siUBE2B. It was found that the expression of UBE2B was suppressed after KYSE-150/ECA-109 was transfected with siUBE2B (**Figure 8A**). Subsequently, CCK-8 assay and colony formation assay revealed a significant inhibition in the proliferation of both the cell lines upon UBE2B knockdown (∗p < 0.05,∗∗p < 0.01, ∗∗∗p < 0.001; **Figures 8B**, **9A**). Moreover, the UBE2B-knockdown cells demonstrated decreased migration ability (**Figure 9B**). These findings suggested that UBE2B could be an oncogene promoting cell proliferation and migration of ESCA cells.

## 4 Discussion

Research has provided evidence that genes responsible for regulating ubiquitination enzymes are implicated in the development of different types of cancers, including ESCA. A prime illustration of this is the E3 ligase, such as NF168, which has been determined to intensify the aggressiveness of ESCA by bolstering the stability of STAT1 (23). Additionally, HECTD3 has been identified as a facilitator of ESCA cell growth and survival by inhibiting the activation of caspase-9 (24). Furthermore, in ESCA tissues, E2 enzymes like UBE2C are found to be significantly overexpressed, and their levels are associated with unfavorable survival outcomes for ESCA patients (25). Indeed, based on the identified roles of ubiquitination-related enzymes in ESCA initiation and progression, targeting these enzymes could potentially be a promising strategy for treating ESCA. In this study, the emphasis was on investigating the role of UBE2B in ESCA. UBE2B is a gene that is highly expressed in ESCA tissues and was found to have a significant correlation with the prognosis of ESCA patients, as indicated by its high P value and HR. However, few studies systematically analyzed the expression of UBE2B and its specific function in ESCA.

UBE2B, an evolutionarily conserved enzyme among eukaryotes, interacted with RAD18 to play a pivotal role in post-replicative DNA repair (26). In addition, UBE2B directly affects the tolerance and repair of DNA damage by its involvement in modifying chromatin through the ubiquitination of histone 2B, which leads to changes in chromatin structure and facilitates the recruitment of DNA repair factors (27–29). Studies reported that UBE2B acted as a positive regulator of the canonical Wnt signaling pathway by affecting the stability and activity of β-catenin, promoting cancer development (30). In addition to its involvement in the progression of oral squamous cancer, UBE2B also played a role in enhancing the cisplatin resistance of triple-negative breast cancer (31). Consequently, the activation of UBE2B leads to the enhancement of stem cell gene expression and promotion of malignant characteristics in ovarian cells, thereby contributing to chemotherapy resistance, metastasis, and recurrence in patients with ovarian cancer (32). As such, targeting UBE2B may present a promising therapeutic approach for the prevention and treatment of various cancers. This research demonstrates that UBE2B functions as an oncogene, driving the aggressive growth and migration of ESCA cells, while also serving as a significant prognostic marker for patients with ESCA. However, the specific E3 ligases that interact with UBE2B in ESCA and contribute to its role in tumor development are not yet fully understood. Further investigation is needed to better confirm and understand the molecular mechanisms underlying UBE2B-mediated ESCA development.

Bioinformatic analyses have revealed that UBE2B expression levels vary across different clinical groups, with a significant increase observed in overweight patients with ESCA. This finding is noteworthy as previous studies have established a correlation between high weight, increased ESCA risk, and adverse survival outcomes (3). Furthermore, our findings show that UBE2B expression levels are significantly elevated in patients with ESCC and are strongly correlated with poor prognosis in ESCC patients. However, no such association was observed in patients with esophageal adenocarcinoma ESAD. Additionally, UBE2B demonstrated promising potential as a predictive and diagnostic biomarker for ESCA, independently predicting patient outcomes. Despite establishing a relationship between UBE2B expression and clinical data in ESCA patients, it is important to note that further investigations are necessary to validate these results. This could involve larger patient cohorts, prospective studies, and functional experiments to elucidate the underlying correlation between UBE2B expression and clinical information of patients with ESCA.

The nuclear factor-κB (NF-κB) pathway plays a role in promoting proliferation and angiogenesis in multiple types of human cancers, among other signaling pathways involved in oncogenesis (33).. Resistance to chemotherapeutic drugs in ESCC is observed in the NF-κB pathway, which not only enhances proliferation, angiogenesis, and metastasis, but also contributes to the development of ESCC (34). In addition, tumor necrosis factor-alpha (TNF-α) has been implicated in the development of ESCC from precancerous to cancerous lesions (35). TNF-α activates the NF-κB pathway, leading to the initiation of inflammation, which in turn contributes to the progression of ESCC (36). By suppressing the body’s anti-tumor immune response, inflammatory reactions contribute significantly to the progression and spread of cancer. Moreover, these reactions have the potential to enhance cancer resistance through the activation of tumor stem cells (37). Thus, previous research has utilized inflammatory response biomarkers as individual factors to anticipate the survival prognosis of patients with ESCA (38, 39). On the other hand, inflammatory cytokines can instigate EMT, an essential mechanism that initiates invasion and metastasis of ESCA (40). Inhibiting ESCA progression has been regarded as an effective strategy by targeting EMT. Additionally, it has been observed that hypoxia disrupts the oxidative stress equilibrium in normal cells, resulting in therapeutic resistance and cancer progression (41). Hypoxia-related genes modulate cellular metabolism and molecular responses by regulating the EMT transcription factors/repressors and EMT-related signaling pathways (42, 43). In ESCC, HIF-2α has been found to promote the proliferation and migration of tumor cells by inducing the process of EMT through the activation of the NOTCH signaling pathway (44). In this study, it was intriguingly observed that these signaling pathways and biological processes that promote the aggressive behavior of tumor cells were enriched specifically in patients with ESCA who had high expression of UBE2B. Considering the involvement of UBE2B in driving the malignant progression of ESCA by modulating these pathways and processes, it is possible that UBE2B plays a role in the progression of ESCA and contributes to poor prognosis.

In ESCA, the number of tumor-infiltrating macrophages and T cells are higher than those in other solid tumors (45). Although it was found that high expression of UBA1 increased the level of CD8 T cells and macrophages, the level of Tem decreased. As a member of memory T cell, Tem have high levels of receptors to mediate strong immediate effector function to response the inflammation, which also participates in anti-tumor (46). Therefore, UBA1 might help tumor immune escape by suppressing Tem. Additionally, it was found that the expression of UBA1 was negatively correlated with the level of Th17 cells. Th17 cells were mainly reported in autoimmune diseases in previous studies, but now they are found in many cancer types like melanoma, ovarian cancer, and colorectal cancer (47). However, the role of Th17 cells in cancers seemed to be contradictory. One study compared the impact of IL-17 and Th17 cells on cancer patient survival, and found that high Th17 cell frequencies improved the survival outcomes, whereas high IL-17 contributed to poor prognosis (48). Therefore, whether UBA1 promote the development by inhibiting the number of Th17 cells in ESCA needs further researches.

In summary, this study aimed to elucidate the specific functions and mechanisms of UBE2B in ESCA development and progression, potentially identifying it as a novel therapeutic target or prognostic marker. By focusing on UBE2B, the researchers aimed to gain insights into its potential role in ESCA biology and its significance in patient outcomes.

## Data availability statement

The original contributions presented in the study are included in the article/**Supplementary Material**. Further inquiries can be directed to the corresponding authors.

## Author contributions

HD: Data curation, Formal analysis, Validation, Writing – original draft, Writing – review & editing. J-CX: Formal analysis, Visualization, Writing – review & editing. Z-GD: Validation, Writing – review & editing. L-FW: Software, Writing – review & editing. Y-BL: Visualization, Writing – review & editing. Y-FZ: Visualization, Writing – review & editing. T-YC: Conceptualization, Methodology, Writing – review & editing. Y-QZ: Conceptualization, Funding acquisition, Writing – review & editing. P-HZ: Conceptualization, Project administration, Supervision, Writing – original draft, Writing – review & editing.

## Supplementary material

Supplementary Table 1 The list of genes associated with the prognosis of patients with ESCA.
